# Black-White Gap Across Levels of Educational Childhood Opportunities: Findings from the ABCD Study

**DOI:** 10.31586/ojer.2024.1124

**Published:** 2024-11-05

**Authors:** Shervin Assari, Hossein Zare

**Affiliations:** 1 Department of Internal Medicine, Charles R. Drew University of Medicine and Science, Los Angeles, CA, United States; 2 Department of Family Medicine, Charles R. Drew University of Medicine and Science, Los Angeles, CA, United States; 3 Department of Urban Public Health, Charles R. Drew University of Medicine and Science, Los Angeles, CA, United States; 4 Marginalization-Related Diminished Returns (MDRs) Center, Los Angeles, CA, United States; 5 Department of Health Policy and Management, Johns Hopkins Bloomberg School of Public Health, Baltimore, MD, United States; 6 School of Business, University of Maryland Global Campus (UMGC), Adelphi, United States

**Keywords:** Racial Disparities, Educational Outcomes, School Discrimination, School Discipline, Childhood Opportunity Index, Academic Achievement, Black-White Achievement Gap

## Abstract

**Objective:**

This study examines racial disparities in educational outcomes—including reading proficiency, grade point average (GPA), school discrimination, and school disciplinary actions—across regions with different levels of educational childhood opportunity index (COI). Our aim is to explore how these racial gaps between Black and White students vary in areas with differing educational opportunities. We hypothesize that higher COI is associated with smaller academic achievement gaps but may also correspond with greater racial bias in unfair school treatment.

**Methods:**

Data were drawn from the Adolescent Brain Cognitive Development (ABCD) study, which provides comprehensive measures of educational outcomes, cognitive performance, and COI. National COI rankings were used to classify regions into five categories: very high, high, average, low, and very low educational opportunity. We analyzed racial gaps in reading proficiency, and experiences of discrimination and suspension across these COI categories. Multi-group Structural Equation Models (SEM) were used to assess how the relationship between race and educational outcomes varies across COI levels.

**Results:**

Our findings confirmed that Black-White gaps in reading proficiency and cognitive test performance (Flanker task) were less pronounced in regions with higher COI. However, racial disparities in school disciplinary actions and experiences of discrimination were more pronounced in higher-opportunity areas. Specifically, the effect of Black race was stronger in regions with the highest COI, where Black students experienced a disproportionately higher rate of unfair school treatment, including both school discrimination and suspensions, compared to their White peers.

**Conclusion:**

This exploratory study supports that while higher educational opportunities are associated with smaller academic achievement gaps between Black and White students, they might be linked to increased racial bias in school disciplinary actions and discriminatory treatment. These findings underscore the complexity of educational equity, suggesting that improving access to quality education alone is insufficient to eliminate racial disparities in school experiences. Addressing school-based bias and discrimination must accompany efforts to enhance educational opportunities.

## 1. Introduction

Racial disparities in educational outcomes—such as reading proficiency, grade point average (GPA), and the frequency of school disciplinary actions—persist across generations in the United States. These disparities contribute to significant inequalities in college admissions, which serve as a critical gateway to economic success and social mobility. Scholars have explored various factors contributing to these gaps, with some emphasizing the role of family background, while others highlight potential genetic factors. However, the influence of schooling and the broader school environment is undeniable [1,2].

School quality and educational opportunities are among the most critical factors shaping cognitive development and future economic outcomes [1]. The racial gap in access to high-quality schools is a persistent issue, exacerbating inequalities in educational attainment. Unfair treatment in schools—whether in the form of discrimination or disproportionate disciplinary actions such as suspensions—also plays a significant role in the Black-White achievement gap. These forms of systemic bias further hinder Black students’ academic progress and long-term success.

While educational opportunities for Black children are often constrained by structural factors, some Black families live in areas with better schools and more favorable educational environments. However, it remains unclear whether the Black-White achievement gap, cognitive performance disparities, or experiences of unfair treatment (discrimination and suspension) differ across regions with varying levels of educational opportunity [5].

Recent efforts have emerged to assess educational opportunities on a national scale, enabling researchers to compare racial disparities in achievement, cognitive development, and school-based discrimination across regions with different levels of childhood opportunity. This study aims to explore these variations in racial gaps by examining achievement, cognitive performance, and instances of unfair treatment in areas with low versus high levels of educational childhood opportunity index (COI) [3,4].

Our hypothesis is that as educational opportunities increase, the cognitive and academic achievement gaps between Black and White students will diminish. However, these improved educational environments may also exhibit increased biases against Black students, potentially resulting in higher rates of unfair treatment such as discrimination and suspension. Thus, this study seeks to investigate the complexities of how educational opportunity shapes both achievement gaps and the experiences of racial bias in school settings.

## 2. Methods

### 2.1. Setting and Design

This study utilized data from the Adolescent Brain Cognitive Development (ABCD) study, a large-scale, longitudinal dataset designed to explore the factors influencing children’s brain development, cognitive functioning, and educational outcomes. The ABCD study is nationally representative, drawing from a diverse population of children across the United States. It collects comprehensive information on participants’ family socioeconomic status (SES), neighborhood characteristics, academic performance, and neuroimaging data related to brain development. The ABCD data is particularly well-suited for examining how environmental and contextual factors—such as educational opportunity—shape cognitive and educational outcomes over time.

### 2.2. Measures

#### 2.2.1. Moderator (Strata):

##### Educational Opportunity Levels:

Educational opportunity levels were measured using residential data that capture local school quality, the availability of educational resources, and access to academic enrichment opportunities in the neighborhood. This measure is based on the Childhood Opportunity Index (COI) available in the ABCD dataset, which reflects the broader social and educational context in which children are raised. Higher COI values indicate greater access to high-quality educational opportunities. For this analysis, COI was treated as a five-level ordinal variable, categorized into very high, high, average, low, and very low educational opportunity.

#### 2.2.2. Predictor

##### Race:

Race was self-reported by the participants or their guardians and was dichotomized into Black and White for the purposes of this study. This dichotomization was necessary to explore specific racial disparities in educational outcomes and school experiences.

#### 2.2.3. Outcomes

##### Flanker Task Performance:

We measured cognitive control using the NIH Toolbox Flanker Inhibitory Control and Attention Test, age-corrected. This task assesses a participant’s ability to focus on a central stimulus while inhibiting responses to surrounding distracting stimuli. The resulting score is a continuous variable, where higher scores indicate better cognitive function, reflecting greater attentional control and inhibition.

##### Reading Ability:

Reading ability was assessed using the NIH Toolbox Reading Recognition Test, age-corrected to account for developmental differences across participants. This test measures a student’s ability to recognize and comprehend written words. Like the Flanker task, the reading ability score is a continuous variable, with higher scores reflecting greater reading proficiency.

##### Perceived School Discrimination:

Perceived school discrimination was assessed through student self-reports using a series of items designed to capture discriminatory experiences in the school setting. These items included questions about unfair treatment by teachers or peers based on race or ethnicity. The responses were averaged to create a continuous measure of perceived school discrimination, where higher scores indicate greater experiences of discrimination.

##### School Discipline (Suspension):

School disciplinary actions were measured using a self-reported item that asked students whether they had ever been suspended from school. This variable was binary, with responses coded as 1 for students who had been suspended and 0 for those who had not. This measure captures an important aspect of school disciplinary actions often associated with racial disparities.

##### Depression (Withdrawal):

Depression symptoms were measured using the depression and withdrawal subscale from the Child Behavior Checklist (CBC). This subscale provides a continuous measure of depressive symptoms, where higher scores indicate greater levels of depression and withdrawal behaviors. The CBC is widely used in research for assessing emotional and behavioral problems in children and adolescents.

#### 2.2.4. Covariates

Several covariates were included to control for other factors that might influence educational outcomes:

##### Child’s age:

Continuous variable to account for developmental differences.

##### Gender:

Dichotomized as male and female.

##### Parental marital status:

Included to account for family structure, which may influence educational experiences and outcomes.

### 2.3. Statistical Analysis

We employed Structural Equation Modeling (SEM) to investigate the associations between race and educational outcomes across the five levels of childhood educational opportunity. SEM allows for the simultaneous modeling of multiple relationships between variables, making it ideal for examining how race, as a predictor, interacts with other factors like COI. The primary focus was on the coefficient for race (coded as Black = 1, White = 0) to quantify the racial disparities in educational outcomes (e.g., reading proficiency, Flanker cognitive performance), emotional outcome (depressed), and treatments (e.g., school suspensions, discrimination).

We conducted multigroup SEM analyses, where educational opportunity levels (based on COI)0 defined the groups. This approach allowed us to explore whether the relationship between race and educational outcomes differed across regions with varying levels of educational opportunity.

### 2.4. Ethical Considerations

The ABCD Study adhered to rigorous ethical standards and was approved by the institutional review boards (IRBs) of all participating research sites. Parents or legal guardians provided informed consent for their children’s participation, and the children themselves provided assent. All data were de-identified to ensure participant privacy and confidentiality throughout the research process.

## 3. Results

Table 1 provides the descriptive statistics for the key study variables. The average age of the children in the sample was 9.48 years (SE = 0.005), with a 95% confidence interval ranging from 9.47 to 9.49. Cognitive performance, as measured by the Flanker task, had a mean score of 95.59 (SE = 0.136), and reading ability, adjusted for age, had a mean score of 103.07 (SE = 0.192). Depression levels, represented as Z-scores, had a mean of −0.014 (SE = 0.010), indicating slightly below-average depression symptoms. The mean level of perceived school discrimination was 1.281 (SE = 0.006), with a confidence interval between 1.268 and 1.293.

In terms of race, 72.9% of the sample identified as White (SE = 0.006), and 27.1% identified as Black (SE = 0.006). When broken down by educational childhood opportunity index (COI) levels, 17.1% of the children lived in the lowest opportunity areas, 11.8% in low opportunity areas, 15.3% in average opportunity areas, 21.7% in high opportunity areas, and 34.1% in the highest opportunity areas.

The sample was fairly balanced by gender, with 47.6% of participants identifying as female (SE = 0.007) and 52.4% as male (SE = 0.007). Regarding the marital status of the household, 34.0% of children lived in an unwed household (SE = 0.006), while 66.0% lived in a married household (SE = 0.006).

Finally, the majority of children had not been suspended from school, with 93.7% reporting no suspension (SE = 0.003), and 6.3% having experienced suspension (SE = 0.003).

The correlation matrix presented in Table 2 shows significant associations among the key study variables. Being Black (Race) was significantly correlated with lower cognitive function, as measured by the Flanker task (r = −0.128, p < 0.05), and with lower reading scores (r = −0.211, p < 0.05). Black children were also significantly more likely to experience school suspension (r = 0.215, p < 0.05) and report higher levels of perceived school discrimination (r = 0.167, p < 0.05). Age was not significantly correlated with race (r = −0.008, p > 0.05), but was positively associated with a higher likelihood of school suspension (r = 0.042, p < 0.05). Male children were more likely to face school suspension (r = 0.133, p < 0.05) and perceive higher school discrimination (r = 0.090, p < 0.05). Living in a married household was associated with better cognitive function (r = 0.094, p < 0.05), higher reading scores (r = 0.206, p < 0.05), and lower rates of both perceived school discrimination (r = −0.117, p < 0.05) and school suspension (r = −0.183, p < 0.05). Depression was not significantly associated with race (r = 0.038, p < 0.05), but was positively correlated with school suspension (r = 0.130, p < 0.05) and school discrimination (r = 0.078, p < 0.05).

**Figure 1.**
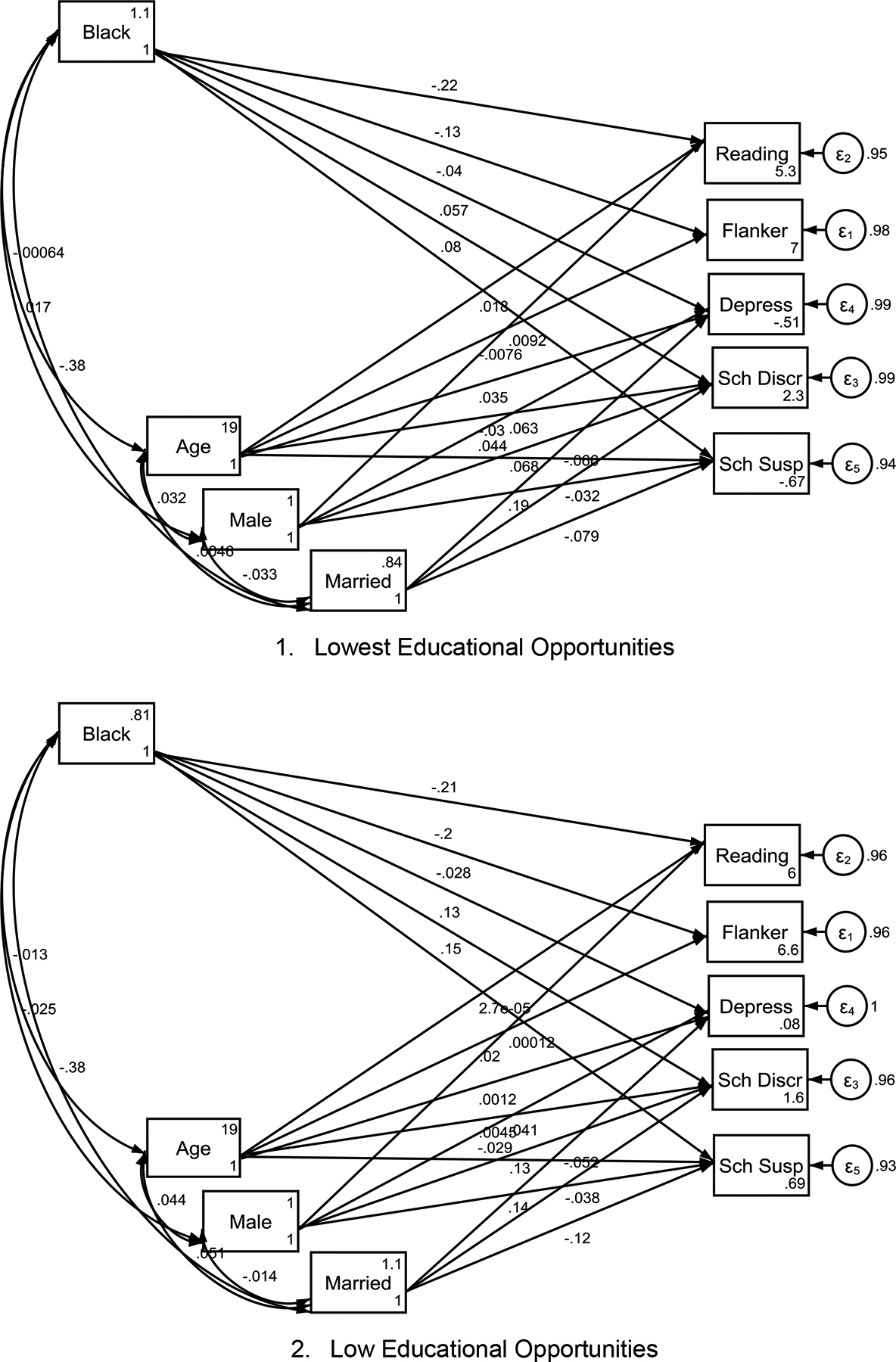

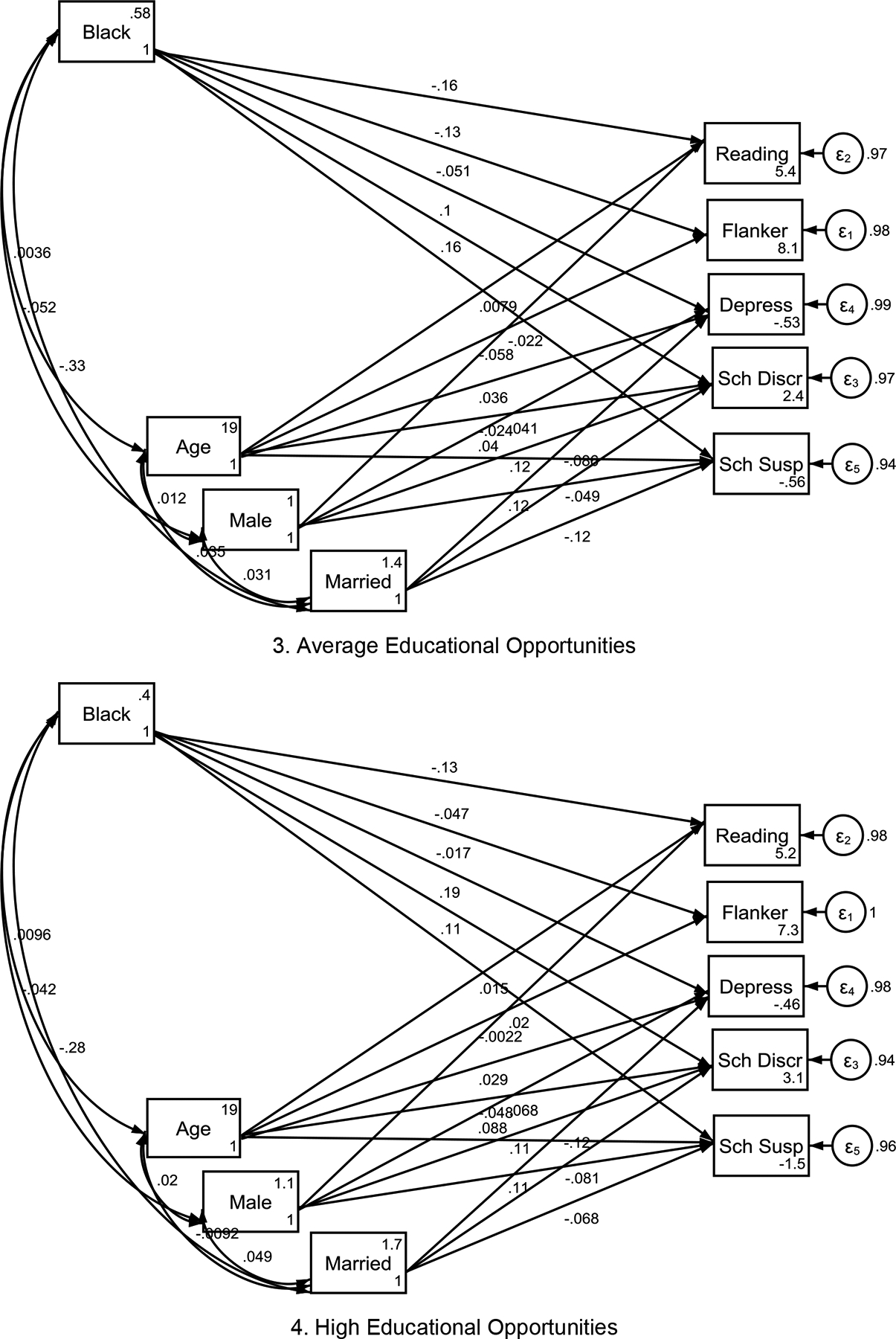

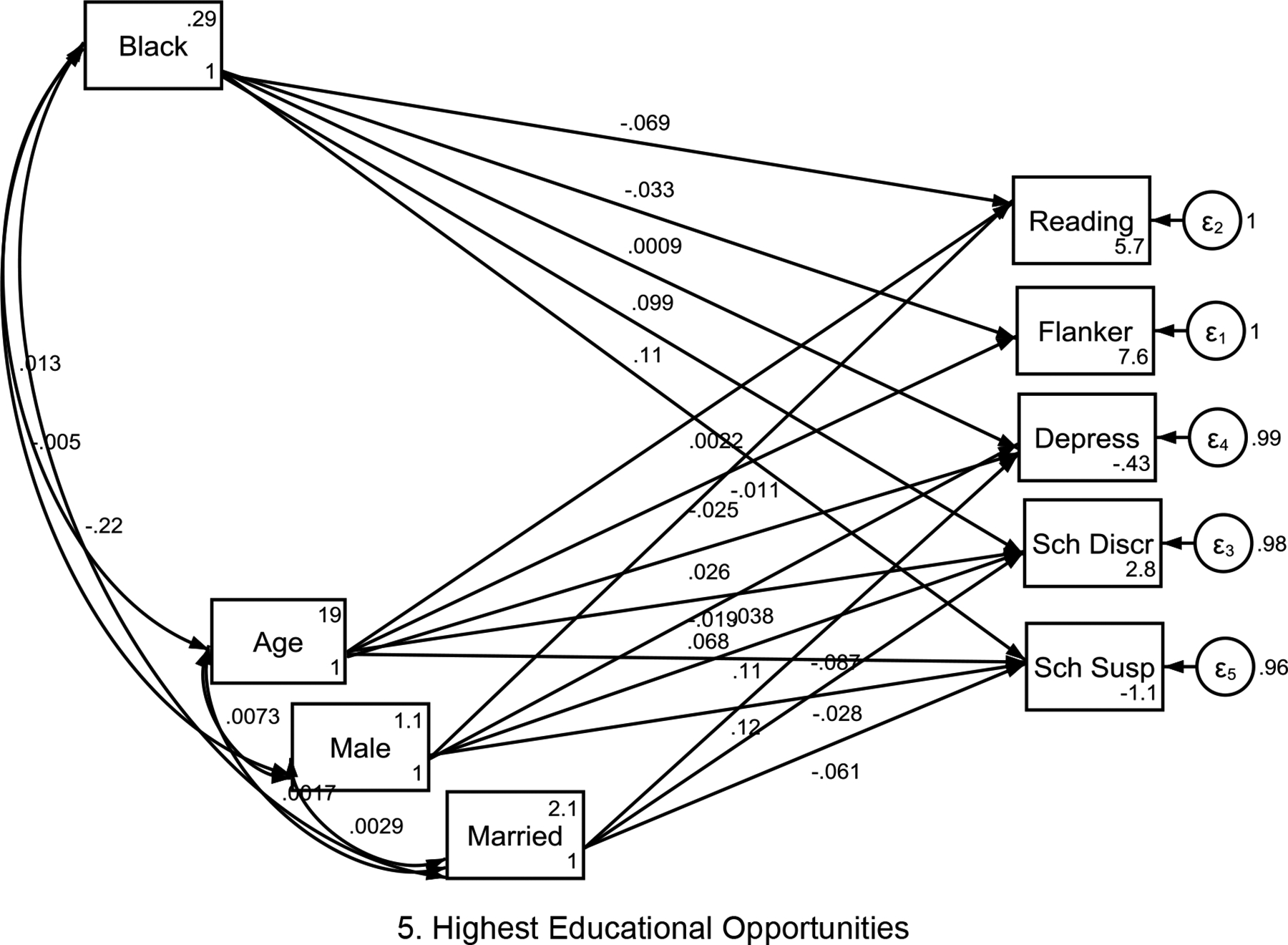
Racial gap in reading proficiency, and experiences of discrimination and suspension across childhood opportunity index (COI) categories

Table 3 presents a summary of the five Structural Equation Models (SEMs) across groups defined by different levels of childhood educational opportunity (COI). Figure 1-1 to 1-5 show the models.

### Lowest Educational COI:

In the lowest educational opportunity areas, being Black was significantly associated with poorer cognitive function (B = −0.125, p < 0.001) and lower reading scores (B = −0.218, p < 0.001). Black children had a slightly higher likelihood of being suspended from school (B = 0.080, p = 0.017). However, Black students did not report significantly higher perceptions of school discrimination compared to White children (B = 0.057, p = 0.062). There was no significant association between race and depression (B = −0.040, p = 0.167).

### Low Educational COI:

In areas with low educational opportunity, Black children showed significantly lower cognitive function (B = −0.196, p < 0.001) and reading scores (B = −0.205, p < 0.001) than White children. Black children also reported significantly higher perceived school discrimination (B = 0.127, p < 0.001) and were more likely to be suspended from school (B = 0.149, p < 0.001). There was no significant association between race and depression (B = −0.028, p = 0.381).

### Average Educational COI:

In areas with average educational opportunities, Black children had significantly lower cognitive function (B = −0.130, p < 0.001) and reading scores (B = −0.158, p < 0.001). They also experienced significantly higher levels of perceived school discrimination (B = 0.103, p < 0.001) and were more likely to be suspended from school (B = 0.157, p < 0.001). There was no significant association between race and depression (B = −0.051, p = 0.062).

### High Educational COI:

In high educational opportunity areas, Black children showed slightly lower cognitive function (B = −0.047, p = 0.023) and significantly lower reading scores (B = −0.128, p < 0.001). Black students also reported significantly higher perceived school discrimination (B = 0.189, p < 0.001) and were more likely to be suspended from school (B = 0.109, p < 0.001). Race was not significantly associated with depression (B = −0.017, p = 0.423).

### Highest Educational COI:

In the highest educational opportunity areas, Black children did not exhibit significantly lower cognitive function (B = −0.033, p = 0.058) but had slightly lower reading scores (B = −0.069, p < 0.001) than White children. However, they were considerably more likely to face school suspension (B = 0.113, p < 0.001) and reported significantly higher perceived school discrimination (B = 0.099, p < 0.001). There was no significant association between race and depression (B = 0.001, p = 0.959).

## 4. Discussion

This study set out to examine the Black-White achievement gap across regions with varying levels of educational childhood opportunity index (COI), focusing on both academic outcomes and experiences of school-based discrimination. We hypothesized that higher COI would be associated with smaller gaps in academic achievement but would also exacerbate racial disparities in school discipline and discrimination. The findings confirmed this dual pattern: while academic achievement gaps in reading proficiency and cognitive performance narrowed in regions with higher COI, racial disparities in school discipline and experiences of unfair treatment widened in these same regions.

The first key takeaway from our analysis is that higher educational opportunity is indeed associated with a reduced Black-White gap in reading proficiency and cognitive performance. This supports prior research indicating that better schooling environments can mitigate some of the racial inequalities in educational outcomes by providing Black students with access to higher-quality resources, teachers, and academic support. However, the persistence of even small achievement gaps, despite improvements in COI [3], suggests that structural inequalities beyond school quality, such as differential access to out-of-school learning resources and lingering effects of early childhood disadvantages, may continue to affect Black students’ educational outcomes. Thus, while increasing educational opportunities is a crucial step toward reducing racial disparities, it does not fully close the achievement gap.

A more concerning finding from this study is that the regions with higher COI also showed more pronounced racial disparities in school disciplinary actions and experiences of discrimination. Black students in these higher-opportunity areas faced disproportionately higher rates of suspensions and reported more instances of unfair treatment compared to their White peers. These results align with research on the Marginalization-Related Diminished Returns (MDRs) framework, which posits that Black individuals may experience fewer returns on their socioeconomic resources, such as access to high-quality education, due to systemic bias and discrimination [5]. This suggests that even when Black students are placed in educational environments that should theoretically foster their success [2,3], they are still vulnerable to racially biased treatment that can undermine their academic and socio-emotional well-being [5].

The finding that higher COI is associated with increased school discrimination and suspension rates for Black students presents an important challenge for policymakers and educators aiming to promote educational equity. It suggests that merely improving educational opportunities without addressing underlying racial biases in school environments may not be enough to ensure equal treatment and outcomes for Black students [5]. Anti-racist training for educators, reforms in school disciplinary policies, and greater accountability for discriminatory practices are critical to creating school environments where all students can thrive, regardless of race.

These results are also indicative of “Minority Diminished Returns” (MDRs [5]). Diminished returns of family socioeconomic status for Black families, known as “Minority Diminished Returns” (MDRs [5]) is supported by evidence showing that Black children often do not benefit from socioeconomic resources to the same extent as their White counterparts. Despite achieving similar levels of parental education and income, Black families frequently face structural barriers such as residential segregation, discrimination in housing and employment, and underfunded schools in predominantly Black neighborhoods [6–13]. These systemic inequalities reduce the impact of family SES on educational opportunities. For example, research has shown that in affluent neighborhoods, Black families encounter discrimination and isolation [14–16]. Additionally, systemic discrimination in school systems, including disparities in disciplinary practices and tracking, further limits Black children’s access to the full range of educational opportunities, undermining the potential benefits of parental education and income [17]. These structural factors contribute to the weaker association between family SES and educational outcomes for Black children, highlighting the need for policies that address racial inequities at multiple levels, from housing to school funding.

In addition to diminished educational opportunities, our findings may explain why we observe weaker cognitive and neurodevelopmental benefits typically associated with higher SES for Black than White children [18]. This aligns with previous research showing that Black children from high SES backgrounds often exhibit weaker brain development outcomes compared to their White peers [19–23]. Factors such as chronic stress, exposure to trauma, and limited access to mental health resources may contribute to this discrepancy, reducing the impact of parental education and income on Black children’s brain development.

### 4.1. Implications

The findings of this study have several key implications for educational policy and practice. First, they underscore the importance of increasing access to high-quality educational opportunities as a means of reducing racial achievement gaps. However, the results also highlight the need for a more comprehensive approach that addresses the racial biases embedded within school systems. Policies aimed at promoting educational equity should not only focus on enhancing access to educational resources but must also tackle the systemic issues of racial discrimination and disproportionate school discipline. Without addressing these biases, even the best-resourced schools may fail to provide equitable educational experiences for Black students. Furthermore, these findings suggest that interventions such as bias training for educators and reforms in school disciplinary policies are essential components of any strategy aimed at closing the racial achievement gap.

### 4.2. Limitations

Despite the robust dataset from the ABCD study and the advanced modeling techniques used, this research has several limitations. First, the cross-sectional nature of the data limits our ability to establish causal relationships between COI and racial disparities in adolescents’ outcomes. Longitudinal data would provide a clearer picture of how these disparities evolve over time and whether changes in educational opportunity or school policies influence the observed outcomes. Second, our reliance on COI rankings as a measure of educational opportunity may oversimplify the complex factors that contribute to school quality and student success. Future research could incorporate more nuanced measures of school environments, including teacher quality, curriculum diversity, and peer support. Lastly, the study does not account for potential variation in individual student experiences within the same regions, which may be influenced by factors such as socioeconomic status, neighborhood segregation, or parental involvement.

### 4.3. Next Research Steps

Future research should aim to build on these findings by addressing the limitations mentioned above. Longitudinal studies that track students over time could offer deeper insights into the causal relationships between educational opportunities, racial disparities, and student outcomes. Additionally, future studies should explore the intersection of race with other variables such as gender, immigration status, and socioeconomic background to understand how different forms of marginalization interact with educational opportunities and bias. Researchers should also examine specific school policies and practices—such as zero-tolerance discipline policies and school-level anti-racism initiatives—that may influence racial disparities in disciplinary actions and discrimination. Finally, future work could investigate interventions designed to reduce school-based discrimination and disciplinary disparities, assessing their effectiveness in reducing the racial gap in both academic achievement and experiences of unfair treatment. By expanding our understanding of these dynamics, future research can contribute to more comprehensive strategies for promoting racial equity in education.

## 5. Conclusion

In conclusion, while higher educational COIs reduce some aspects of the Black-White achievement gap, they also expose Black students to greater risks of unfair treatment within schools. This duality highlights the complex nature of educational inequality in the United States. To fully address these disparities, interventions must go beyond improving access to educational opportunities and focus on transforming the school culture to eliminate racial bias and discriminatory practices.

## Figures and Tables

**Table 1.** Descriptive Data Overall

	Mean	Std. err.	95% conf.	interval
age	9.480	0.005	9.470	9.490
Flanker (Age Corrected)	95.590	0.136	95.322	95.857
Reading (Age Corrected)	103.065	0.192	102.688	103.442
Depression (Z score)	−0.014	0.010	−0.033	0.006
Perceived School Discrimination	1.281	0.006	1.268	1.293
	%	SE	[95% conf.	interval]
Race				
White	0.729	0.006	0.717	0.740
Black	0.271	0.006	0.260	0.283
Educational Childhood Opportunity Index (COI)				
Lowest	0.171	0.005	0.161	0.182
Low	0.118	0.004	0.110	0.127
Average	0.153	0.005	0.144	0.163
High	0.217	0.006	0.206	0.228
Highest	0.341	0.006	0.328	0.353
Gender				
Female	0.476	0.007	0.463	0.489
Male	0.524	0.007	0.511	0.537
Marital Status of the Household				
Unwed Household	0.340	0.006	0.327	0.352
Married Household	0.660	0.006	0.648	0.673
School Suspension				
No	0.937	0.003	0.931	0.944
Yes	0.063	0.003	0.056	0.069

**Table 2.** Pearson Correlation between Study Variables

	1	2	3	4	5	6	7	8	9
1 Race (Black)	1.000								
2 Age	−0.008	1.000							
3 Male	−0.021*	0.019	1.000						
4 Married Household	−0.408*	0.023*	0.010	1.000					
5 Flanker	−0.128*	−0.023*	0.031*	0.094*	1.000				
6 Reading	−0.211*	0.015	0.014	0.206*	0.198*	1.000			
7 Depression	0.038*	0.025*	0.055*	−0.103*	−0.040*	−0.025*	1.000		
8 School Discrimination	0.167*	−0.031*	0.090*	−0.117*	−0.063*	−0.141*	0.078*	1.000	
9 School Suspension	0.215*	0.042*	0.133*	−0.183*	−0.066*	−0.108*	0.130*	0.114*	1.000

* p<0.05

**Table 3.** Summary of structural equation model (SEM) across educational childhood opportunity index (COI) levels

			B	SE	95%	CI	p
Lowest Educational COI							
Age (Year)	→	Cognitive Function (Flanker)	−0.008	0.024	−0.056	0.040	0.757
Race (Black)	→	Cognitive Function (Flanker)	−0.125	0.026	−0.176	−0.074	< 0.001
Intercept	→	Cognitive Function (Flanker)	6.991	0.470	6.070	7.911	< 0.001
Age (Year)	→	Reading Score	0.018	0.024	−0.029	0.065	0.457
Gender (Male)	→	Reading Score	0.009	0.024	−0.038	0.057	0.703
Race (Black)	→	Reading Score	−0.218	0.025	−0.268	−0.169	< 0.001
Intercept	→	Reading Score	5.312	0.461	4.409	6.216	< 0.001
Age (Year)	→	Perceived School Discrimination	−0.030	0.026	−0.082	0.021	0.251
Gender (Male)	→	Perceived School Discrimination	0.068	0.026	0.017	0.119	0.009
Family Structure (Married Family)	→	Perceived School Discrimination	−0.032	0.028	−0.088	0.023	0.255
Race (Black)	→	Perceived School Discrimination	0.057	0.031	−0.003	0.117	0.062
Intercept	→	Perceived School Discrimination	2.262	0.490	1.301	3.223	< 0.001
Age (Year)	→	Depression	0.035	0.024	−0.013	0.083	0.152
Gender (Male)	→	Depression	0.063	0.024	0.015	0.111	0.010
Family Structure (Married Family)	→	Depression	−0.066	0.027	−0.118	−0.013	0.014
Race (Black)	→	Depression	−0.040	0.029	−0.096	0.017	0.167
Intercept	→	Depression	−0.506	0.456	−1.400	0.387	0.267
Age (Year)	→	School Suspension	0.044	0.030	−0.014	0.102	0.141
Gender (Male)	→	School Suspension	0.189	0.029	0.132	0.246	< 0.001
Family Structure (Married Family)	→	School Suspension	−0.079	0.033	−0.144	−0.015	0.016
Race (Black)	→	School Suspension	0.080	0.034	0.014	0.146	0.017
Intercept	→	School Suspension	−0.668	0.554	−1.753	0.418	0.228
Low Educational COI							
Age (Year)	→	Cognitive Function (Flanker)	0.020	0.028	−0.035	0.075	0.470
Race (Black)	→	Cognitive Function (Flanker)	−0.196	0.029	−0.253	−0.139	< 0.001
Intercept	→	Cognitive Function (Flanker)	6.642	0.540	5.584	7.699	< 0.001
Age (Year)	→	Reading Score	0.000	0.028	−0.055	0.055	0.999
Gender (Male)	→	Reading Score	0.000	0.028	−0.055	0.055	0.996
Race (Black)	→	Reading Score	−0.205	0.029	−0.262	−0.149	< 0.001
Intercept	→	Reading Score	5.994	0.533	4.950	7.038	< 0.001
Age (Year)	→	Perceived School Discrimination	0.005	0.029	−0.053	0.062	0.878
Gender (Male)	→	Perceived School Discrimination	0.127	0.029	0.069	0.184	< 0.001
Family Structure (Married Family)	→	Perceived School Discrimination	−0.038	0.032	−0.102	0.025	0.235
Race (Black)	→	Perceived School Discrimination	0.127	0.034	0.061	0.193	< 0.001
Intercept	→	Perceived School Discrimination	1.551	0.548	0.476	2.625	0.005
Age (Year)	→	Depression	0.001	0.028	−0.054	0.057	0.966
Gender (Male)	→	Depression	0.041	0.028	−0.014	0.096	0.147
Family Structure (Married Family)	→	Depression	−0.052	0.031	−0.112	0.009	0.093
Race (Black)	→	Depression	−0.028	0.032	−0.092	0.035	0.381
Intercept	→	Depression	0.080	0.525	−0.949	1.109	0.878
Age (Year)	→	School Suspension	−0.029	0.035	−0.098	0.040	0.411
Gender (Male)	→	School Suspension	0.142	0.035	0.074	0.211	< 0.001
Family Structure (Married Family)	→	School Suspension	−0.121	0.038	−0.195	−0.047	0.001
Race (Black)	→	School Suspension	0.149	0.038	0.075	0.223	< 0.001
Intercept	→	School Suspension	0.690	0.654	−0.591	1.971	0.291
Average Educational COI							
Age (Year)	→	Cognitive Function (Flanker)	−0.058	0.024	−0.106	−0.011	0.015
Race (Black)	→	Cognitive Function (Flanker)	−0.130	0.025	−0.179	−0.082	< 0.001
Intercept	→	Cognitive Function (Flanker)	8.133	0.463	7.226	9.041	< 0.001
Age (Year)	→	Reading Score	0.008	0.024	−0.039	0.055	0.743
Gender (Male)	→	Reading Score	−0.022	0.024	−0.070	0.025	0.353
Race (Black)	→	Reading Score	−0.158	0.025	−0.206	−0.109	< 0.001
Intercept	→	Reading Score	5.422	0.463	4.515	6.329	< 0.001
Age (Year)	→	Perceived School Discrimination	−0.024	0.025	−0.073	0.025	0.331
Gender (Male)	→	Perceived School Discrimination	0.115	0.025	0.067	0.164	< 0.001
Family Structure (Married Family)	→	Perceived School Discrimination	−0.049	0.027	−0.101	0.003	0.065
Race (Black)	→	Perceived School Discrimination	0.103	0.028	0.048	0.157	< 0.001
Intercept	→	Perceived School Discrimination	2.356	0.467	1.440	3.272	< 0.001
Age (Year)	→	Depression	0.036	0.024	−0.012	0.083	0.142
Gender (Male)	→	Depression	0.041	0.024	−0.007	0.088	0.094
Family Structure (Married Family)	→	Depression	−0.086	0.026	−0.137	−0.036	0.001
Race (Black)	→	Depression	−0.051	0.027	−0.104	0.003	0.062
Intercept	→	Depression	−0.528	0.454	−1.418	0.362	0.245
Age (Year)	→	School Suspension	0.040	0.032	−0.023	0.103	0.215
Gender (Male)	→	School Suspension	0.124	0.032	0.061	0.186	< 0.001
Family Structure (Married Family)	→	School Suspension	−0.118	0.034	−0.185	−0.050	0.001
Race (Black)	→	School Suspension	0.157	0.033	0.091	0.222	< 0.001
Intercept	→	School Suspension	−0.562	0.602	−1.742	0.619	0.351
High Educational COI							
Age (Year)	→	Cognitive Function (Flanker)	−0.002	0.020	−0.042	0.037	0.913
Race (Black)	→	Cognitive Function (Flanker)	−0.047	0.021	−0.088	−0.007	0.023
Intercept	→	Cognitive Function (Flanker)	7.308	0.393	6.539	8.078	< 0.001
Age (Year)	→	Reading Score	0.015	0.020	−0.024	0.054	0.454
Gender (Male)	→	Reading Score	0.020	0.020	−0.019	0.059	0.313
Race (Black)	→	Reading Score	−0.128	0.020	−0.168	−0.088	< 0.001
Intercept	→	Reading Score	5.175	0.385	4.420	5.930	< 0.001
Age (Year)	→	Perceived School Discrimination	−0.048	0.020	−0.087	−0.009	0.017
Gender (Male)	→	Perceived School Discrimination	0.105	0.020	0.066	0.145	0.000
Family Structure (Married Family)	→	Perceived School Discrimination	−0.081	0.021	−0.122	−0.040	0.000
Race (Black)	→	Perceived School Discrimination	0.189	0.022	0.146	0.232	0.000
Intercept	→	Perceived School Discrimination	3.111	0.380	2.366	3.857	0.000
Age (Year)	→	Depression	0.029	0.020	−0.009	0.068	0.138
Gender (Male)	→	Depression	0.068	0.020	0.030	0.107	0.001
Family Structure (Married Family)	→	Depression	−0.115	0.021	−0.156	−0.075	0.000
Race (Black)	→	Depression	−0.017	0.021	−0.059	0.025	0.423
Intercept	→	Depression	−0.455	0.376	−1.193	0.283	0.227
Age (Year)	→	School Suspension	0.088	0.028	0.034	0.142	0.002
Gender (Male)	→	School Suspension	0.107	0.027	0.053	0.160	0.000
Family Structure (Married Family)	→	School Suspension	−0.068	0.029	−0.124	−0.011	0.019
Race (Black)	→	School Suspension	0.109	0.028	0.055	0.163	0.000
Intercept	→	School Suspension	−1.489	0.523	−2.513	−0.464	0.004
Highest Educational COI							
Age (Year)	→	Cognitive Function (Flanker)	−0.025	0.017	−0.058	0.007	0.131
Race (Black)	→	Cognitive Function (Flanker)	−0.033	0.017	−0.067	0.001	0.058
Intercept	→	Cognitive Function (Flanker)	7.597	0.321	6.968	8.227	< 0.001
Age (Year)	→	Reading Score	0.002	0.017	−0.030	0.035	0.893
Gender (Male)	→	Reading Score	−0.011	0.017	−0.044	0.021	0.489
Race (Black)	→	Reading Score	−0.069	0.017	−0.103	−0.035	< 0.001
Intercept	→	Reading Score	5.652	0.319	5.026	6.278	< 0.001
Age (Year)	→	Perceived School Discrimination	−0.019	0.017	−0.052	0.013	0.247
Gender (Male)	→	Perceived School Discrimination	0.113	0.017	0.081	0.146	< 0.001
Family Structure (Married Family)	→	Perceived School Discrimination	−0.028	0.017	−0.062	0.006	0.108
Race (Black)	→	Perceived School Discrimination	0.099	0.018	0.063	0.134	< 0.001
Intercept	→	Perceived School Discrimination	2.790	0.318	2.167	3.413	< 0.001
Age (Year)	→	Depression	0.026	0.016	−0.006	0.058	0.114
Gender (Male)	→	Depression	0.038	0.016	0.006	0.070	0.022
Family Structure (Married Family)	→	Depression	−0.087	0.017	−0.120	−0.054	< 0.001
Race (Black)	→	Depression	0.001	0.018	−0.033	0.035	0.959
Intercept	→	Depression	−0.434	0.311	−1.043	0.176	0.163
Age (Year)	→	School Suspension	0.068	0.022	0.025	0.111	0.002
Gender (Male)	→	School Suspension	0.121	0.022	0.078	0.163	< 0.001
Family Structure (Married Family)	→	School Suspension	−0.061	0.022	−0.105	−0.017	0.007
Race (Black)	→	School Suspension	0.113	0.022	0.070	0.157	< 0.001
Intercept	→	School Suspension	−1.144	0.414	−1.956	−0.332	0.006
